# Longitudinal associations of serum MCP-1 with cognitive decline and psychological stress in biomarker-confirmed Alzheimer's disease: A pilot study

**DOI:** 10.1016/j.bbih.2026.101333

**Published:** 2026-08-22

**Authors:** Fumihiko Yasuno, Yasuyuki Kimura, Yumeka Yamauchi, Hiroyuki Minami, Kensaku Kasuga, Takeshi Ikeuchi, Akinori Takeda, Takashi Sakurai

**Affiliations:** a National Hospital for Geriatric Medicine, National Center for Geriatrics and Gerontology, Obu, Japan; b Department of Clinical and Experimental Neuroimaging, Center for Development of Advanced Medicine for Dementia, National Center for Geriatrics and Gerontology, Obu, Japan; c Equipment Management Division, Center for Core Facility Administration, National Center for Geriatrics and Gerontology, Obu, Japan; d Department of Diagnostic Innovation Science, National Center for Geriatrics and Gerontology, Obu, Japan; e Department of Molecular Genetics, Brain Research Institute, Niigata University, Niigata, Japan

**Keywords:** Monocyte chemoattractant protein-1, Alzheimer's disease, Neuroinflammation, Cognitive decline, Psychological stress, Glial cell activation

## Abstract

**Introduction:**

Monocyte chemoattractant protein-1 (MCP-1; also known as CCL2) is a chemokine implicated in neuroinflammatory pathways that may contribute to cognitive decline in Alzheimer's disease (AD). Elevated MCP-1 is associated with glial activation, blood-brain barrier dysfunction, and immune-cell recruitment. This study assessed the longitudinal association between peripheral MCP-1 and cognitive decline in biomarker-confirmed participants across the AD continuum and explored whether psychological stress was associated with longitudinal MCP-1 changes.

**Methods:**

This pilot longitudinal study included 21 amyloid-/p-tau-positive participants across the AD continuum, including six with mild cognitive impairment and 15 with AD. Participants underwent baseline and a single 1-year follow-up assessment. Global cognition was assessed using the Alzheimer's Disease Assessment Scale cognitive subscale Japanese version, and serum MCP-1 concentrations were measured using enzyme-linked immunosorbent assay. Stress response, depression, anxiety, apathy, and neuropsychiatric symptoms were assessed using validated behavioral and psychological scales. Stepwise multiple regression and partial correlation analyses were performed.

**Results:**

Annual changes in peripheral MCP-1 were significantly associated with cognitive decline (β = 0.54, P = 0.005). Psychological stress, measured by the Stress Response Scale-18, was positively correlated with MCP-1 changes (r = 0.75, P = 0.003). These findings suggest that longitudinal serum MCP-1 changes may be associated with cognitive decline in the AD continuum and that psychological stress may be related to MCP-1 dynamics.

**Discussion:**

MCP-1 may contribute to AD-related neuroinflammatory activity. The association between psychological stress and MCP-1 changes should be interpreted as exploratory and requires validation in larger longitudinal cohorts.

## Highlights

• Serum MCP-1 changes were linked to cognitive decline in this pilot AD cohort.
• Psychological stress correlated with longitudinal serum MCP-1 changes.
• Biomarker-confirmed participants across the AD continuum were studied.
• Peripheral MCP-1 may reflect stress-related neuroinflammatory activity.

## Graphical abstract

**Figure:**
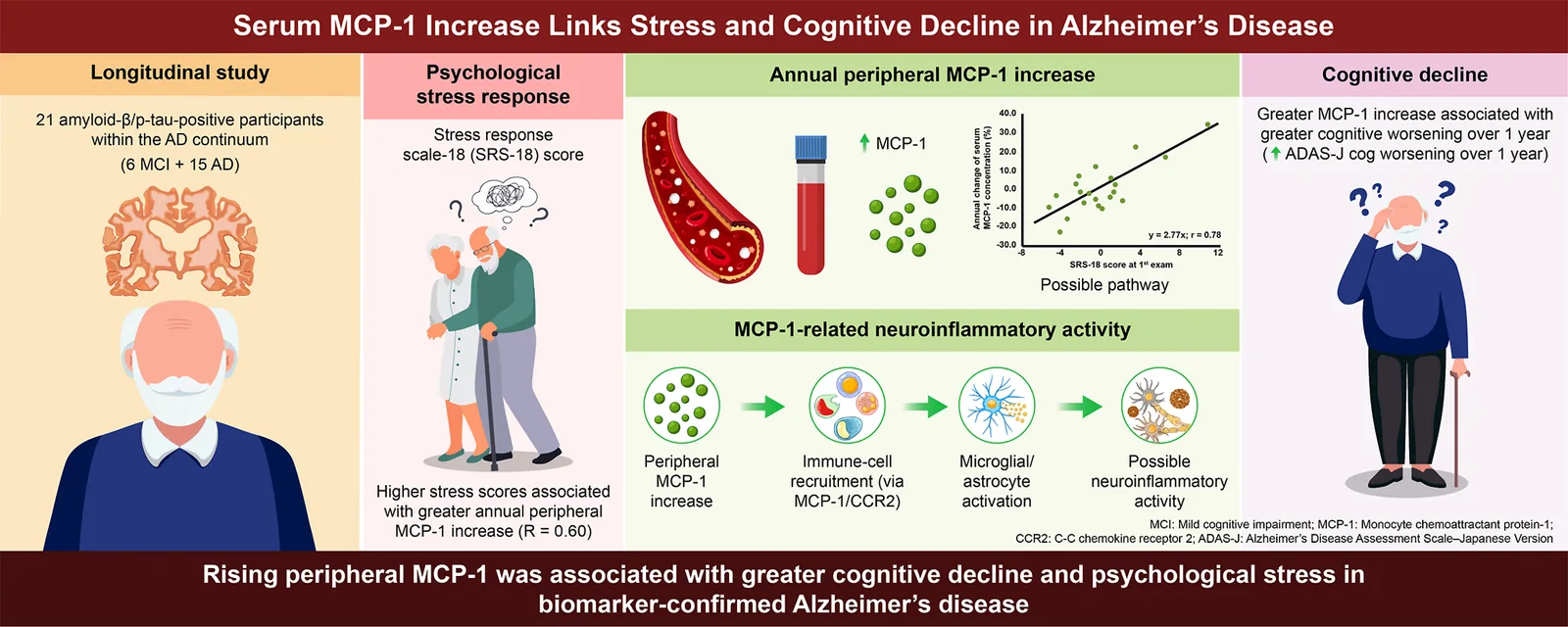

## 1 Introduction

Sustained dysregulation of immune homeostasis, together with prolonged glial activation, has been implicated in cognitive deterioration through neurodegenerative processes, including synaptic damage and neuronal loss (Albornoz et al., 2018). Within this inflammatory environment, activated microglia produce various inflammatory mediators (Park et al., 2020), which may further activate microglia and astrocytes and sustain neuroinflammation (Chen et al., 2015; Elsaafien et al., 2019). Monocyte chemoattractant protein-1 (MCP-1; also known as CCL2) is one such mediator and has attracted attention for its role in neuroimmune dysfunction (Babcock et al., 2003).

MCP-1 guides circulating immune cells, such as monocytes and lymphocytes, toward the central nervous system (CNS) through its interaction with CC-chemokine receptor 2 (CCR2) (Sozzani et al., 1994). CCR2 is expressed on immune-cell surfaces (Boddeke et al., 1999; Mack et al., 2001), and MCP-1/CCR2 signaling has been shown to facilitate immune-cell entry into multiple tissues, including the brain (Babcock et al., 2003; D'Mello et al., 2009; Izikson et al., 2000). This pathway has also been implicated in several neuroinflammatory disorders, including AD (Galimberti et al., 2006).

Increased MCP-1 expression has been reported in AD brains and transgenic animal models of AD. Cytokine and chemokine profiling of brain tissue from patients with AD has shown higher MCP-1 levels than in age-matched controls (Ishizuka et al., 1997; Sokolova et al., 2009). Moreover, it has been shown that CSF MCP-1 levels were associated with the transition from mild cognitive impairment (MCI) to AD (Galimberti et al., 2006). Mechanistic studies indicate that Aβ-activated glial cells may release MCP-1, which can contribute to blood-brain barrier (BBB) dysfunction and facilitate immune-cell recruitment into the CNS (El Khoury et al., 2003; Roberts et al., 2012; Smits et al., 2002). These observations raise the possibility that peripheral immune alterations may, at least in part, reflect neuroinflammatory processes occurring in the CNS (Britschgi and Wyss-Coray, 2007; Sun et al., 2003). Because MCP-1 is involved in immune-cell migration and CNS immune-cell trafficking, its measurement in peripheral blood may offer insight into neuroinflammatory activity (Sozzani et al., 1994).

Collectively, prior findings suggest that MCP-1 may be involved in AD-related neuroinflammatory processes by facilitating immune-cell recruitment to sites of neural injury and thereby sustaining inflammatory activity. Evidence from animal models (Villeda et al., 2011; Villeda and Wyss-Coray, 2013) and human cohorts (Bettcher et al., 2016, 2019; Kimura et al., 2018; Lee et al., 2018; Westin et al., 2012) has linked elevated MCP-1 levels in CSF or blood with functional decline and structural brain changes associated with cognitive deterioration in older adults. Nevertheless, most human evidence comes from cross-sectional studies, healthy aging cohorts, or clinically defined AD samples without biomarker confirmation.

In this pilot longitudinal study, we investigated whether annual changes in serum MCP-1 were associated with cognitive decline in biomarker-confirmed participants across the AD continuum. Because MCP-1 has been implicated in inflammatory responses relevant to AD pathology, we focused primarily on the association between longitudinal MCP-1 dynamics and annual cognitive change. In addition, given prior evidence linking psychological stress to peripheral inflammatory responses, we explored whether behavioral and psychological measures, including psychological stress, were associated with annual changes in serum MCP-1.

## 2 Material and methods

### 2.1 Study participants

This single-center pilot longitudinal study included 21 amyloid-/p-tau-positive participants across the AD continuum and examined changes in cognitive function and serum MCP-1 levels between baseline and a single follow-up assessment conducted 1 year later. The cohort comprised six individuals with MCI and 15 with AD. Global cognition was assessed at baseline using the Mini-Mental State Examination (MMSE) and Alzheimer's Disease Assessment Scale cognitive subscale Japanese version (ADAS-J cog). The MMSE is a brief 30-point screening measure of global cognitive function, with lower scores indicating greater cognitive impairment (Folstein et al., 1975). The ADAS-J cog is widely used in clinical studies to evaluate cognitive domains, including memory, orientation, visuospatial function, language, and praxis (Rosen et al., 1984). Dementia severity was assessed using the Clinical Dementia Rating (CDR), a standardized instrument for staging AD-related dementia (Morris, 1993).

MCI was diagnosed based on clinical assessment of memory impairment, a CDR score of 0.5, and largely preserved daily functioning, together with relatively preserved global cognition as indicated by an MMSE score of 24–30. The MMSE score range was not used by itself to define objective memory impairment. Probable AD was diagnosed according to the National Institute on Aging–Alzheimer's Association criteria (McKhann et al., 2011) with an MMSE score of <24. Raw MMSE scores were used in the present study and were not adjusted for educational attainment.

Serum and cerebrospinal fluid (CSF) samples were collected and stored at −80°C in the National Center for Geriatrics and Gerontology (NCGG) biobank. Amyloid and phosphorylated tau (p-tau181) status were determined using the CSF Aβ42/40 ratio and p-tau181 concentrations measured at the Brain Research Institute of Niigata University. Positivity thresholds were defined as an Aβ42/40 ratio of <0.072 (Kasuga et al., 2023) and a p-tau181 concentration of >30.6 pg/mL (Kasuga et al., 2022), based on reference values from the Japanese Alzheimer's Disease Neuroimaging Initiative. APOE4 genotype was determined by polymerase chain reaction analysis of DNA extracted from participant samples (Hixson and Vernier, 1990). Participants carrying at least one ε4 allele were classified as APOE4-positive. After baseline assessment, 16 participants started donepezil therapy at 5 mg/day.

The study protocol was reviewed and approved by the Institutional Review Board of the NCGG (Approval Nos. 1276-5 and 1507). Written informed consent was obtained from all participants before study enrollment.

### 2.2 Assessment of annual cognitive changes and serum MCP-1 concentration

Global cognition was assessed using the ADAS-J cog at baseline and at the 1-year follow-up assessment, and annual score changes were calculated for each participant. Blood samples for serum preparation were collected at baseline and at the 1-year follow-up assessment. A total of 24 mL of whole blood was centrifuged at 2350 × g at 4°C for 5 and 10 min. Serum was aliquoted into 1.5-mL tubes at 0.3 mL per tube and stored at −80°C in the NCGG biobank until analysis. Serum MCP-1 concentrations were determined using enzyme-linked immunosorbent assay kits (R&D Systems, Inc., Minneapolis, MN, USA; Catalog No. DPC00), according to the manufacturer's protocol. All serum samples were analyzed in duplicate, and the mean value of the duplicate measurements was used for statistical analysis. To minimize inter-assay variability, paired baseline and follow-up samples from each participant were analyzed using the same ELISA kit and in the same assay run. Optical density was measured using a SpectraMax Paradigm microplate reader (Molecular Devices, LLC, San Jose, CA, USA). Annual percentage changes in serum MCP-1 concentrations were then calculated.

### 2.3 Assessment of behavioral and psychological index scores

Depressive symptoms were assessed using the Japanese version of the Self-Rating Depression Scale (SDS), a 20-item self-report questionnaire originally developed by Zung, with each item rated on a four-point Likert scale (Fukuda and Kobayashi, 1973; Zung, 1965). Apathy was assessed using the Japanese version of the Apathy Scale, a translated version of Starkstein's Apathy Scale that has been validated in Japanese patients (Okada et al., 1998; Starkstein et al., 1993). The scale provides total scores ranging from 0 to 42, with higher scores indicating greater apathy.

Anxiety was assessed using the Japanese version of the State-Trait Anxiety Inventory (STAI), which includes separate 20-item subscales for state anxiety (STAI-S) and trait anxiety (STAI-T), both rated on a four-point Likert scale (Iwata and Mishima, 1999; Spielberger et al., 1983). Raw STAI scores were converted to T-scores according to the scoring procedures and normative data provided for the Japanese version of the STAI. Neuropsychiatric symptoms were assessed using the Japanese version of the 12-item Neuropsychiatric Inventory Questionnaire (NPI-Q), which has been validated in Japanese patients with dementia (Kaufer et al., 2000; Matsumoto et al., 2006). Each item was scored from 0 to 3, with higher scores indicating greater symptom severity.

Psychological stress was assessed as an exposure-related factor using the Stress Response Scale-18 (SRS-18), a Japanese instrument developed and validated to assess daily stress responses (Suzuki et al., 1997). The scale includes 18 items, including “I feel anxious,” “I feel sad,” “I feel short-tempered,” and “I have difficulty concentrating,” rated from 0 (“not at all”) to 3 (“true”). Higher scores indicate stronger stress responses. Raw SRS-18 scores were converted to T-scores according to the standardized scoring procedure for the Japanese population. Previous studies have reported good internal consistency in general populations, with Cronbach's alpha values of 0.82–0.88.

### 2.4 Statistical analysis

Baseline demographic, clinical, biomarker, and behavioral/psychological measures are summarized using median [interquartile range] for continuous variables and n (%) for categorical variables. For descriptive comparisons between participants with MCI and those with AD, continuous variables were compared using the Mann–Whitney *U* test, and categorical variables were compared using Fisher's exact test owing to the small sample size.

Exploratory multiple linear regression analyses were performed to examine factors associated with annual cognitive change. The dependent variable was annual change in ADAS-J cog score. Candidate explanatory variables included age, sex, diagnosis (MCI vs. AD), APOE4 genotype, initiation of anti-dementia medication after baseline assessment, CSF Aβ42/40 ratio, CSF p-tau181 concentration, baseline ADAS-J cog score, baseline serum MCP-1 concentration, and annual percentage change in serum MCP-1 concentration.

As a sensitivity analysis, we examined whether the association between annual percentage change in serum MCP-1 concentration and annual change in ADAS-J cog score was consistent when the analysis was restricted to participants with AD. Owing to the small number of AD participants, this subgroup analysis was performed using Spearman's rank correlation rather than multivariable regression.

Given the pilot design and small sample size, the analyses were treated as exploratory rather than confirmatory. A stepwise variable-selection method was used to derive a parsimonious final exploratory model. All candidate explanatory variables were initially considered for entry into the model, and variables were sequentially entered or removed according to the probability-of-F criteria implemented in SPSS. The entry criterion was set at P < 0.05, and the removal criterion was set at P > 0.10. Variables retained in this model were interpreted as correlates of annual cognitive change rather than definitive predictors. The Shapiro–Wilk test was used to assess data normality, and Box–Cox transformations were applied to the CSF Aβ42/40 ratio and CSF p-tau181 values.

To examine whether APOE4 status or initiation of anti-dementia medication was associated with serum MCP-1 levels or longitudinal MCP-1 changes, exploratory post hoc analyses were performed. Baseline serum MCP-1 concentrations and annual percentage changes in serum MCP-1 concentrations were compared according to APOE4 status and initiation of anti-dementia medication after baseline assessment using the Mann–Whitney *U* test.

After the association between serum MCP-1 change and cognitive change was identified, partial correlation analyses were performed to explore relationships between annual MCP-1 change and behavioral or psychological index scores. These analyses controlled for age, sex, baseline ADAS-J cog score, APOE4 status, initiation of anti-dementia medication, CSF Aβ42/40 ratio, and CSF p-tau181 concentration.

Analyses were performed using SPSS for Windows, version 26.0 (IBM Corp., Armonk, NY, USA). All tests were two-tailed, and Bonferroni-corrected significance was set at P < 0.05/n, where n represents the number of comparisons.

## 3 Results

### 3.1 Baseline participant characteristics and longitudinal changes in cognition and serum MCP-1

**Fig. 1:**
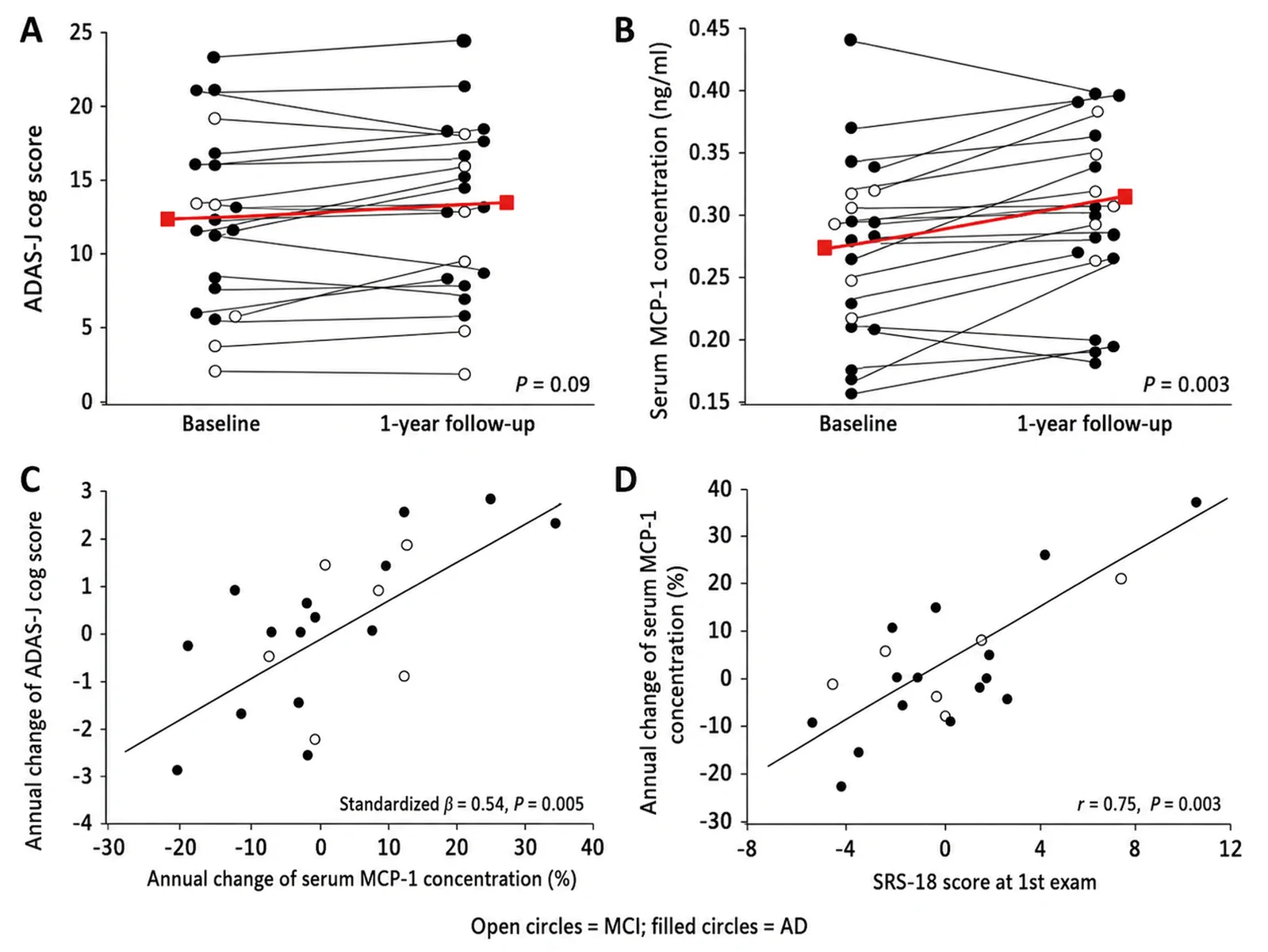
**Longitudinal changes in cognitive function and serum MCP-1 concentration, and their associations with psychological stress across the AD continuum**. (A) Individual ADAS-J cog scores at baseline and 1-year follow-up. (B) Individual serum MCP-1 concentrations at baseline and 1-year follow-up. In panels A and B, open circles indicate participants with MCI and filled circles indicate participants with AD. Red squares connected by a red line indicate mean values at each time point. P values from paired t-tests are shown in the panels. (C) Partial residual plot showing the association between annual change in ADAS-J cog score and annual percentage change in serum MCP-1 concentration after adjustment for variables included in the final exploratory model. (D) Partial residual plot showing the association between annual percentage change in serum MCP-1 concentration and SRS-18 score after adjustment for clinical and pathological covariates. In panels C and D, open circles indicate participants with MCI and filled circles indicate participants with AD. Abbreviations: AD, Alzheimer's disease; ADAS-J cog, Alzheimer's Disease Assessment Scale–Japanese Cognitive Subscale; MCI, mild cognitive impairment; MCP-1, monocyte chemoattractant protein-1; SRS-18, Stress Response Scale-18. (For interpretation of the references to colour in this figure legend, the reader is referred to the Web version of this article.)

Baseline demographic, clinical, biomarker, and behavioral/psychological characteristics are summarized in Table 1. The cohort consisted of 21 participants, including six with MCI and 15 with AD. The median age of the total cohort was 77.0 years, and 13 participants were female. The median baseline MMSE score was 23.0, and the median baseline ADAS-J cog score was 13.3, indicating that the cohort mainly included participants with MCI or mild-to-moderate AD. Twelve participants were APOE4-positive, and 16 participants initiated anti-dementia medication after baseline assessment. Behavioral and psychological measures, including SDS, Apathy Scale, STAI-S, STAI-T, NPI-Q, and SRS-18 scores, are also shown in Table 1. Comparisons between the MCI and AD groups were performed to examine diagnostic subgroup differences. Baseline MMSE scores were significantly lower in participants with AD than in those with MCI, reflecting greater global cognitive impairment in the AD group. In contrast, behavioral and psychological measures did not differ significantly between the MCI and AD groups. Because of the small number of participants in each diagnostic subgroup, these comparisons should be interpreted cautiously. During the 1-year follow-up, ADAS-J cog scores showed a nonsignificant trend toward an increase (t = 1.78, P = 0.09), whereas serum MCP-1 concentrations increased significantly (t = 3.42, P = 0.003; Fig. 1A and B).

**Table 1:** Participant characteristics, cognitive scores, behavioral and psychological measures, and serum MCP-1 measurements across the AD continuum.

Characteristic/Test	Total patients	MCI patients	AD patients	P-value
No.	21	6	15	-
Female sex, n (%)	13 (61.9)	3 (50.0)	10 (66.7)	0.48
Age, y, median (IQR)	77.0 (6.0)	81.5 (7.0)	76.0 (5.0)	0.13
MMSE, median (IQR)	23.0 (3.5)	26.0 (4.3)	21.0 (3.0)	<0.001*
ADAS-J-cog score, median (IQR)	13.3 (8.9)	10.7 (10.5)	13.3 (7.8)	0.27
ADAS-J-cog score, median (IQR) (1 year later)	14.2 (9.1)	12.2 (11.1)	15.3 (9.1)	0.27
Annual change of ADAS-J-cog score, median (IQR)	0.3 (2.1)	0.4 (2.8)	0.3 (1.6)	1.00
Behavioral and psychological measures				
SDS score, median (IQR)	30.0 (8.0)	29.0 (11.8)	30.0 (7.0)	0.68
Apathy Scale score, median (IQR)	13.0 (6.5)	9.0 (13.8)	13.0 (4.0)	0.24
STAI-S T-score, median (IQR)	39.5 (9.8)	37.5 (21.5)	40.0 (9.3)	0.72
STAI-T T-score, median (IQR)	45.0 (8.0)	48.0 (21.5)	44.5 (13.3)	0.55
NPI-Q score, median (IQR)	2.0 (3.5)	1.5 (3.0)	2.0 (4.0)	0.47
SRS-18 T-score, median (IQR)	43.0 (7.0)	45.0 (7.8)	43.0 (6.0)	0.42
Serum MCP-1 concentration (pg/mL), median (IQR)	293 (98.0)	307.0 (72.2)	289.1 (123.1)	0.57
Serum MCP-1 concentration (pg/mL), median (IQR) (1 year later)	308.3 (85.5)	320.8 (61.1)	294.7 (154.9)	0.57
Annual change of MCP-1 (%), median (IQR)	7.1 (17.1)	12.4 (14.4)	6.0 (19.3)	0.38
CSF Aβ42/40 ratio values, median (IQR)	0.05 (0.04)	0.03 (0.04)	0.05 (0.02)	0.79
CSF p-tau 181, median (IQR)	44.6 (27.5)	46.1 (51.2)	44.6 (22.5)	0.73
Initiation of anti-dementia drugs, n (%)	16 (76.2)	4 (66.7)	12 (80.0)	0.52
APOE4-positive, n (%)	12 (57.1)	3 (50.0)	9 (60.0)	0.68

*p < 0.05.

Data are presented as median (interquartile range) for continuous variables and n (%) for categorical variables. P values were calculated using the Mann–Whitney *U* test for continuous variables and Fisher's exact test for categorical variables. APOE4-positive was defined as carrying at least one ε4 allele.

Abbreviations: AD, Alzheimer's disease; ADAS-J cog, Alzheimer's Disease Assessment Scale–Japanese Cognitive Subscale; APOE4, apolipoprotein E ε4; CSF, cerebrospinal fluid; IQR, interquartile range; MCP-1, monocyte chemoattractant protein-1; MCI, mild cognitive impairment; MMSE, Mini-Mental State Examination; NPI-Q, Neuropsychiatric Inventory Questionnaire; SDS, Self-Rating Depression Scale; SRS-18, Stress Response Scale-18; STAI-S, State-Trait Anxiety Inventory-State; STAI-T, State-Trait Anxiety Inventory-Trait.

### 3.2 Association between annual MCP-1 changes and cognitive decline

Exploratory multiple linear regression analysis was used to identify variables associated with annual changes in the ADAS-J cog scores using the stepwise variable-selection procedure described in Section 2.4. The full exploratory model, including all candidate explanatory variables, is shown in Supplementary Table 1. The final model retained initiation of anti-dementia medication and annual percentage change in MCP-1 levels as variables associated with annual ADAS-J cog change (Table 2). Age, sex, diagnosis, APOE4 status, baseline serum MCP-1 concentration, baseline ADAS-J cog score, CSF Aβ42/40 ratio, and CSF p-tau181 concentration were not retained.

**Table 2:** Final exploratory multiple linear regression model for annual change in ADAS-J cog score.

Final model	T	Standardized β	β	95% CI	P	VIF
Initiation of anti-dementia drugs	−2.61	−0.44	−1.79	−3.24 to −0.35	0.02*	1.0
Annual change of MCP-1 (%)	3.18	0.54	0.07	0.02 to 0.12	0.005*	1.0

*p < 0.05.

Candidate explanatory variables were age, sex, diagnosis (MCI vs. AD), APOE4 status, initiation of anti-dementia medication after baseline assessment, CSF Aβ42/40 ratio, CSF p-tau181 concentration, baseline ADAS-J cog score, baseline serum MCP-1 concentration, and annual percentage change in serum MCP-1 concentration. Only variables retained in the final exploratory model are shown in Table 2. The full exploratory model is provided in Supplementary Table 1.

Abbreviations: AD, Alzheimer's disease; ADAS-J cog, Alzheimer's Disease Assessment Scale–Japanese Cognitive Subscale; APOE4, apolipoprotein E ε4; CI, confidence interval; CSF, cerebrospinal fluid; MCP-1, monocyte chemoattractant protein-1; MCI, mild cognitive impairment; VIF, variance inflation factor.

Annual percentage change in MCP-1 levels was positively associated with annual ADAS-J cog change in the final model (unstandardized β = 0.07, 95% CI = 0.02 to 0.12; standardized β = 0.54; t = 3.18; P = 0.005; Fig. 1C). The final model was statistically significant (F = 8.34, df = 2,18; P = 0.003; R^2^ = 0.48). These findings indicate that greater increases in peripheral MCP-1 were associated with greater cognitive decline over one year. Initiation of anti-dementia medication was negatively associated with annual ADAS-J cog change, suggesting a possible association with reduced cognitive decline during follow-up.

In a sensitivity analysis restricted to participants with AD, the association between annual percentage change in serum MCP-1 concentration and annual change in ADAS-J cog score remained statistically significant and directionally consistent with the main analysis (Spearman's ρ = 0.77, P < 0.001). This finding suggests that the main association was not driven solely by the inclusion of participants with MCI, although the subgroup analysis should be interpreted cautiously because of the small number of AD participants.

In exploratory post hoc analyses, baseline serum MCP-1 concentrations did not differ significantly between APOE4-positive and APOE4-negative participants (median [IQR], 287.8 [108.5] vs. 293.0 [114.0] pg/mL; Mann–Whitney U = 53, P = 0.97). Annual percentage changes in serum MCP-1 concentrations also did not differ significantly between APOE4-positive and APOE4-negative participants (median [IQR], 7.4 [19.2] vs. 7.1 [16.5]%; Mann–Whitney U = 48, P = 0.70). Similarly, neither baseline serum MCP-1 concentrations nor annual percentage changes in serum MCP-1 concentrations differed significantly between participants who initiated anti-dementia medication after baseline assessment and those who did not (baseline MCP-1: median [IQR], 297.7 [107.0] vs. 275.4 [104.0] pg/mL; Mann–Whitney U = 30, P = 0.45; annual MCP-1 change: median [IQR], 6.6 [18.0] vs. 7.2 [7.4]%; Mann–Whitney U = 37, P = 0.84).

### 3.3 Partial correlation analysis between annual MCP-1 change and behavioral/psychological measures

After adjustment for clinical and pathological covariates, partial correlation analysis revealed that psychological stress, measured using the SRS-18, was positively associated with annual percentage change in the MCP-1 levels (r = 0.75, P = 0.003; Fig. 1D), which remained significant after Bonferroni correction for six comparisons (corrected threshold, P < 0.0083; Table 3).

**Table 3:** Partial correlation analysis between annual percentage change in MCP-1 and behavioral and psychological index scores.

	Mean ± SD	r	p
SDS score	29.7 ± 4.7	0.45	0.12
Apathy Scale	12.5 ± 5.2	0.03	0.92
STAI state score (T score)	38.8 ± 7.6	0.22	0.49
STAI trait score (T score)	43.2 ± 9.3	0.46	0.13
NPI-Q score	2.2 ± 2.2	−0.21	0.50
SRS-18 score (T score)	43.3 ± 4.6	0.75	0.003*

*P < 0.0083 (0.05/6).

Partial correlation analysis between annual change of MCP-1 (%) and behavioral and psychological index scores with baseline clinical and pathological factors as covariates, including age, sex, ADAS-J cog score, APOE4 positivity, initiation of anti-dementia medication, CSF Aβ42/40 ratio and p-tau181 concentration.

Abbreviations: MCP-1, monocyte chemoattractant protein-1; SD, standard deviation; SDS, Self-Rated Depression Scale ; STAI, State-Trait Anxiety Inventory; NPI-Q, Neuropsychiatric Inventory Questionnaire; SRS-18, Stress Response Scale-18; ADAS-J cog, Alzheimer's Disease Assessment Scale-Japanese Cognitive Subscale; APOE4, apolipoprotein E ε4; CSF, cerebrospinal fluid.

## 4 Discussion

### 4.1 MCP-1 and cognitive deterioration

This pilot longitudinal study examined the relationships among peripheral MCP-1 dynamics, cognitive change, and psychological stress in biomarker-confirmed participants across the AD continuum. The main finding was that greater annual increases in serum MCP-1 were associated with greater worsening of the ADAS-J cog scores over 1 year. In addition, psychological stress was associated with annual MCP-1 change, whereas psychological and behavioral measures were not directly associated with annual cognitive change. Taken together, these findings suggest that longitudinal MCP-1 dynamics may be relevant to cognitive deterioration in AD, while the association between psychological stress and MCP-1 change should be regarded as an exploratory finding. The results are consistent with previous evidence linking MCP-1 to neurodegenerative and neuroinflammatory pathways. Elevated MCP-1 has been observed in AD-related conditions and associated with inflammatory mechanisms involving glial activation and immune-cell recruitment (Galimberti et al., 2006; Izikson et al., 2000; Sokolova et al., 2009). Prior clinical studies have also linked MCP-1 levels in blood or CSF to cognitive impairment, brain atrophy, and longitudinal cognitive decline in older adults and patients with MCI or AD (Bettcher et al., 2016, 2018, 2019; Kimura et al., 2018; Lee et al., 2018; Westin et al., 2012).

Peripheral immune alterations may both reflect and interact with inflammatory processes within the CNS (Britschgi and Wyss-Coray, 2007; Sun et al., 2003). Age- and inflammation-associated BBB disruption may allow circulating immune mediators and immune cells to influence CSF composition and brain parenchymal inflammation (Montagne et al., 2020). Because MCP-1 regulates monocyte migration and CCR2-dependent immune-cell recruitment into the CNS (Babcock et al., 2003; Deshmane et al., 2009; Sozzani et al., 1994), peripheral MCP-1 may serve as a candidate marker of AD-related neuroinflammatory activity associated with cognitive deterioration.

Notably, baseline serum MCP-1 concentration was not retained in the final model, whereas annual MCP-1 change was associated with annual cognitive change. This suggests that longitudinal MCP-1 dynamics, rather than a single baseline measurement, may be more relevant to short-term cognitive deterioration in this pilot cohort. Although previous studies have linked blood or CSF MCP-1 levels to cognitive impairment and longitudinal cognitive decline in MCI and AD, the extent to which a single baseline peripheral MCP-1 measurement predicts subsequent cognitive decline remains uncertain (Bettcher et al., 2016, 2018, 2019; Kimura et al., 2018; Lee et al., 2018; Westin et al., 2012). Repeated MCP-1 assessment may therefore be useful for capturing ongoing inflammatory activity related to cognitive change. However, larger longitudinal cohorts are needed to determine whether MCP-1 changes provide predictive information beyond established AD biomarkers and clinical factors.

### 4.2 Psychological stress and MCP-1

After discussing the association between MCP-1 dynamics and cognitive decline, we considered psychological stress as a possible factor related to peripheral inflammatory changes. Our exploratory analyses revealed that psychological stress, measured using the SRS-18, was associated with annual changes in the peripheral MCP-1 levels. However, because the present study did not include a cognitively normal control group and behavioral and psychological measures were examined in a small sample, this finding should be interpreted cautiously and does not establish temporal or causal relationships.

Importantly, exploratory analyses did not show direct associations between psychological or behavioral measures and annual cognitive change. Therefore, the present findings should not be interpreted as evidence of a direct stress–cognition relationship or as support for a causal pathway from psychological stress to MCP-1 activation and subsequent cognitive decline. Rather, the observed association between psychological stress and MCP-1 dynamics should be regarded as a possible stress-related inflammatory mechanism that warrants further investigation in larger longitudinal cohorts with appropriate control groups.

Previous studies have linked psychological stress to inflammatory biomarkers. Acute and chronic stress have been associated with increased inflammatory activity, including alterations in cytokines and chemokines (Endrighi et al., 2016; Steptoe et al., 2007). Higher peripheral MCP-1 levels have also been reported in relation to psychosocial stress, depressive symptoms, and loneliness (Asberg et al., 2009; Hackett et al., 2012; Suarez et al., 2003). Together, these studies provide a plausible basis for the observed association between SRS-18 scores and MCP-1 changes in this cohort.

Several biological mechanisms may explain the association between psychological stress and inflammatory activation. Psychological stress can increase sympathetic and sympatho-adrenal-medullary activity while reducing parasympathetic regulation (Adams et al., 2004; Cole et al., 2010; Grebe et al., 2010; Slavich and Irwin, 2014). These stress-related pathways may increase nuclear factor-kappa B activity, leading to greater transcription of pro-inflammatory genes and increased production of inflammatory mediators, including chemokines such as MCP-1 (Britschgi and Wyss-Coray, 2007; Slavich and Irwin, 2014). The present study did not directly assess these mechanisms. Future studies with larger samples, appropriate control groups, repeated stress measurements, and broader inflammatory marker panels are needed to determine whether psychological stress is prospectively related to MCP-1 dynamics and cognitive decline in AD.

### 4.3 Clinical implications

From a clinical and translational perspective, the present findings support further investigation of peripheral MCP-1 as a candidate marker related to cognitive decline in the AD continuum. The observed association between psychological stress and MCP-1 dynamics also raises the possibility that stress-related inflammatory responses may be relevant to AD progression. However, these implications remain preliminary and should not be interpreted as evidence that MCP-1 is a clinically useful biomarker or therapeutic target at this stage.

Given its role in monocyte recruitment and inflammatory signaling, the MCP-1/CCR2 pathway has been considered a potential target for therapeutic modulation in neuroinflammatory conditions (Deshmane et al., 2009; Semple et al., 2010; Stone et al., 2017). Therapeutic approaches targeting this pathway, including CCR2 inhibition and RNA interference-based strategies, have been explored in other inflammatory settings (Bégin-Lavallée et al., 2016). However, basal MCP-1/CCR2 signaling also contributes to normal immune defense, indicating that therapeutic modulation would require careful evaluation of efficacy and safety (Deshmane et al., 2009). Accordingly, our findings support further mechanistic and longitudinal research rather than immediate MCP-1-targeted intervention in AD. In parallel, stress-reduction strategies may warrant investigation as low-risk complementary approaches. Whether reducing psychological stress can alter MCP-1-related inflammatory activity or slow cognitive decline in AD remains unknown; therefore, prospective intervention studies are needed before clinical implications can be established.

### 4.4 Strengths and limitations

The present study has several strengths. First, the cohort consisted of biomarker-confirmed amyloid/p-tau-positive individuals across the AD continuum, which strengthens the pathological validity of the findings. Second, the study evaluated longitudinal changes in both cognitive performance and peripheral MCP-1 levels, rather than relying on cross-sectional biomarker associations alone. Third, combining inflammatory biomarker data with psychological stress measures allowed exploration of potential interactions among neuroinflammation, stress-related processes, and cognitive decline.

This study has some limitations. First, the sample size was small, and the study was exploratory; therefore, the findings should not be interpreted as confirmatory, and the regression estimates may be unstable. Second, stepwise variable selection in a small sample may increase the risk of overfitting and model instability, although it was used to derive a parsimonious exploratory model. Third, although MCP-1 and cognitive function were assessed longitudinally, the longitudinal assessment was limited to a single follow-up time point 1 year after baseline. Therefore, the present study could not characterize trajectories of MCP-1 or cognitive change over multiple time points. Moreover, the behavioral and psychological analyses were limited and cannot establish causal or temporal relationships among psychological stress, MCP-1 changes, and cognitive decline. In addition, psychological and behavioral measures were not directly associated with annual cognitive change in this small cohort, and a formal mediation model linking stress, MCP-1 dynamics, and cognition was not tested. Fourth, the single-center cohort included patients with MCI or mild-to-moderate AD, which may limit generalizability. A sensitivity analysis restricted to participants with AD showed a directionally consistent association between annual MCP-1 change and annual cognitive change; however, this subgroup analysis was limited by the small number of AD participants and should be interpreted cautiously. Although APOE4 status and initiation of anti-dementia medication may be relevant to AD pathophysiology and inflammatory biomarker dynamics, our exploratory post hoc analyses did not show significant differences in the baseline serum MCP-1 concentrations or annual MCP-1 changes according to these factors. However, these null findings should be interpreted cautiously owing to the small sample size and should not be taken to exclude potential influences of APOE4 status or anti-dementia medication on longitudinal inflammatory dynamics. Finally, because the analysis was limited to MCP-1, future studies should include broader inflammatory and neurodegenerative biomarker panels to clarify whether these findings are specific to MCP-1.

## 5 Conclusion

In this pilot longitudinal study, greater annual increases in peripheral MCP-1 were associated with greater annual worsening of cognitive performance among individuals within the biomarker-confirmed AD continuum. Psychological stress was also related to annual MCP-1 dynamics, although no direct association between psychological or behavioral measures and cognitive change was observed. These findings require confirmation in larger, independent longitudinal cohorts with appropriate control groups before the role of stress-related inflammatory pathways in AD progression can be established.

## CRediT authorship contribution statement

**Fumihiko Yasuno:** Conceptualization, Formal analysis, Funding acquisition, Investigation, Methodology, Resources, Writing – original draft. **Yasuyuki Kimura:** Data curation, Formal analysis, Investigation, Methodology, Resources, Validation, Writing – review & editing. **Yumeka Yamauchi:** Investigation, Resources, Writing – review & editing. **Hiroyuki Minami:** Investigation, Resources, Writing – review & editing. **Kensaku Kasuga:** Investigation, Resources, Writing – review & editing. **Takeshi Ikeuchi:** Funding acquisition, Investigation, Resources, Writing – review & editing. **Akinori Takeda:** Writing – review & editing. **Takashi Sakurai:** Writing – review & editing.

## Ethics statement

The study protocol was conducted in accordance with the Declaration of Helsinki, relevant laws, and institutional guidelines, and was reviewed and approved by the Institutional Review Board of the National Center for Geriatrics and Gerontology (Approval Nos. 1276-5 and 1507). Written informed consent was obtained from all participants before study enrollment, and the privacy rights of all participants were observed.

## Funding

This work was supported by the 10.13039/100009619Japan Agency for Medical Research and Development [JP23dk0207059 and JP23dm0207073], the 10.13039/501100001691Japan Society for the Promotion of Science [22K07610], and the 10.13039/501100007312National Center for Geriatrics and Gerontology [22-5 and 22-23]. The funders had no role in the study design, collection, analysis, and interpretation of data, writing of the report, or decision to submit the article for publication.

## Declaration of competing interests

The authors declare that they have no known competing financial interests or personal relationships that could have appeared to influence the work reported in this paper.

## Data Availability

The data underlying this study are not publicly available because they contain sensitive participant information and are subject to institutional and ethical restrictions. Data may be made available from the corresponding author upon reasonable request and subject to approval by the relevant institutional ethics committee.
