# Supplementary material for: Longitudinal associations of serum MCP-1 with cognitive decline and psychological stress in biomarker-confirmed Alzheimer's disease: A pilot study
Source: Brain Behav Immun Health. 2026 Aug 22;56:101333. doi: 10.1016/j.bbih.2026.101333 (PMC13543105; doi:10.1016/j.bbih.2026.101333)
Supplement: Supplementary file 1 [file mmc1.docx]

| **Supplemental Table 1. Full exploratory multiple linear regression model for annual change in ADAS-J cog score** | | | | | | |
| --- | --- | --- | --- | --- | --- | --- |
| Step | T | Standardized β | Β | 95% CI | P | VIF |
| Full Model |  |  |  |  |  |  |
| Age | 0.23 | 0.06 | 0.02 | -0.21 to 0.26 | 0.82 | 1.39 |
| Sex | 0.43 | 0.11 | 0.39 | -1.63 to 2.42 | 0.67 | 1.47 |
| Diagnosis (MCI vs AD) | 0.09 | 0.02 | 0.09 | -2.11 to 2.28 | 0.93 | 1.49 |
| APOE4 positivity | -0.31 | -0.08 | -0.26 | -2.17 to 1.65 | 0.76 | 1.36 |
| Initiation of anti-dementia drugs | -1.05 | -0.37 | -1.49 | -4.64 to 1.67 | 0.32 | 2.74 |
| ADAS-J-cog score at 1st exam | 0.11 | 0.03 | 0.01 | -0.18 to 0.20 | 0.91 | 1.58 |
| CSF Aβ42/40 ratio ^†^ | 0.13 | 0.03 | 0.04 | -0.68 to 0.76 | 0.90 | 1.67 |
| CSF p-tau ^†^ | -0.72 | -0.22 | -4.90E+04 | -1.99E+05 to 1.02E+05 | 0.49 | 2.09 |
| Serum MCP-1 at 1st exam | -0.19 | -0.06 | -0.002 | -0.02 to 0.02 | 0.85 | 2.37 |
| Annual change of MCP-1 (%) | 2.14 | 0.53 | 0.07 | -0.003 to 0.14 | 0.06 | 1.39 |
| * p<0.05. | | | | | | |
| ^†^ Data were normalized by Box-Cox transformation | | | | | | |
| The dependent variable was annual change in ADAS-J cog score. β indicates the unstandardized regression coefficient. Candidate explanatory variables were age, sex, diagnosis (MCI vs. AD), APOE4 status, initiation of anti-dementia medication after baseline assessment, CSF Aβ42/40 ratio, CSF p-tau181 concentration, baseline ADAS-J cog score, baseline serum MCP-1 concentration, and annual percentage change in serum MCP-1 concentration. This full exploratory model is provided for transparency; the final exploratory model derived using stepwise variable selection is shown in Table 2. | | | | | | |
| Abbreviations: AD, Alzheimer’s disease; ADAS-J cog, Alzheimer’s Disease Assessment Scale–Japanese Cognitive Subscale; APOE4, apolipoprotein E ε4; CI, confidence interval; CSF, cerebrospinal fluid; MCP-1, monocyte chemoattractant protein-1; MCI, mild cognitive impairment; VIF, variance inflation factor. | | | | | | |
